# Association of Insomnia and Obstructive Sleep Apnea with Worse Oral Mucositis and Quality of Life in Head and Neck Cancer Patients Undergoing Radiation Therapy

**DOI:** 10.3390/cancers16071335

**Published:** 2024-03-29

**Authors:** Austin J. Iovoli, Kelsey Smith, Han Yu, Melissa A. Kluczynski, Carla R. Jungquist, Andrew D. Ray, Mark K. Farrugia, Fangyi Gu, Anurag K. Singh

**Affiliations:** 1 Department of Radiation Medicine, Roswell Park Comprehensive Cancer Center, Elm and Carlton Streets, Buffalo, NY 14263, USA; austin.iovoli@roswellpark.org (A.J.I.); kelsey.bezon@roswellpark.org (K.S.); mark.farrugia@roswellpark.org (M.K.F.); 2 Department of Biostatistics & Bioinformatics, Roswell Park Comprehensive Cancer Center, Elm and Carlton Streets, Buffalo, NY 14263, USA; han.yu@roswellpark.org; 3 Department of Clinical Research Services, Roswell Park Comprehensive Cancer Center, Elm and Carlton Streets, Buffalo, NY 14263, USA; melissa.kluczynski@roswellpark.org; 4 School of Nursing, University at Buffalo, 312 Wende Hall, Buffalo, NY 14214, USA; carlajun@buffalo.edu; 5 Department of Cancer Prevention & Control, Roswell Park Comprehensive Cancer Center, Elm and Carlton Streets, Buffalo, NY 14263, USA; andrew.ray@roswellpark.org; 6 CORDS Oncology, Bristol Myers Squibb, 3401 Princeton Pike, Lawrenceville, NJ 08648, USA; fangyigu@gmail.com

**Keywords:** head and neck cancer, radiation therapy, sleep, oral mucositis, quality of life, insomnia, obstructive sleep apnea

## Abstract

**Simple Summary:**

Patients with head and neck cancer undergoing curative treatment with radiation therapy and chemotherapy often experience sleep disturbances that can affect pain and quality of life. We prospectively studied this patient population using sleep symptom questionnaires to assess the impact of sleep disturbances on patient-reported outcomes. We found that at baseline, 39% of patients had subthreshold or greater insomnia and 54% screened positive for OSA. Upon completion of radiation therapy, patients with these sleep disturbances had worse patient-reported quality of life and oral mucositis pain. These findings highlight the importance of managing sleep disturbances as a potential method to improve patient-reported outcomes for patients undergoing radiation therapy for head and neck cancer.

**Abstract:**

Background: Patients with head and neck cancer (HNC) undergoing radiation therapy (RT) often experience sleep disturbances that may contribute to oral mucositis (OM) and quality of life (QOL). Methods: Patients with HNC treated with RT at a single institution were examined. Sleep questionnaires were given on the first day of RT to assess for insomnia and obstructive sleep apnea (OSA). Patient-reported QOL and oral mucositis were assessed during RT. Associations between insomnia and OSA with QOL were assessed using the Mann–Whitney U test. Linear mixed models assessed associations with OM. Results: Among 87 patients, 34 patients (39%) had subthreshold or greater insomnia and 47 patients (54%) screened positive for OSA. Upon RT completion, patients with subthreshold or greater insomnia had worse physical function (*p* = 0.005), fatigue (*p* = 0.01), insomnia (*p* < 0.001), and sticky saliva (*p* = 0.002). Patients screening positive for OSA had worse physical function (*p* = 0.01), sticky saliva (*p* = 0.02), fatigue (*p* = 0.007), insomnia (*p* = 0.009), and pain (*p* = 0.005). Upon linear mixed model evaluation, subthreshold or greater insomnia (*p* = 0.01) and positive OSA screen (*p* = 0.002) were associated with worse OM. Conclusion: Insomnia and OSA are highly prevalent in patients with HNC undergoing RT. These sleep disturbances are associated with worse QOL and OM during treatment.

## 1. Introduction

Oral mucositis (OM) is a painful, dose-limiting side effect of cancer treatment, occurring in >80% of head and neck cancer (HNC) patients who receive radiation therapy (RT) alone or concurrent chemoradiation therapy (CRT) as treatment [1]. These symptoms often disrupt therapy, increasing the risk of hospitalization and mortality [2].

It has previously been shown that the daily time of radiation treatment significantly impacted OM in HNC patients treated with RT [3]. In a follow-up study, genes relevant to OM were found to have diurnal variation in mRNA expression [4]. The involvement of the circadian rhythm with the development of OM led to our interest in the relationship between sleep and OM pain.

While a reciprocal relationship between sleep and pain has been reported in the general population [5], the relationship between sleep and OM pain in cancer patients has yet to be studied. To explore this knowledge gap, we examined the associations of sleep disturbances with RT-induced OM pain and quality of life (QOL) in HNC patients.

## 2. Materials and Methods

### 2.1. Patient Cohort

The cohort of patients included in the study was derived from 87 patients who were treated with definitive or adjuvant (C)RT for primary HNC at a single institution from August 2018 to October 2021. Patients included in this study had to meet the following eligibility: (1) primary HNC with pathologic confirmation, (2) definitive or adjuvant intent RT treatment received, (3) completed RT, and (4) had sleep questionnaire, quality of life, and oral mucositis data available. Data were collected under a protocol (EDR 103707) with approval by the Roswell Park Comprehensive Cancer Center Institutional Review Board. The Strengthening the Reporting of Observational Studies in Epidemiology (STROBE) recommendations were followed.

### 2.2. Clinical Workup and Patient Treatment

All patients completed staging workup with neck and maxillofacial computed tomography (CT) imaging with contrast and/or positron emission tomography-computed tomography (PET/CT). Radiation therapy was delivered to all patients with intensity modulated radiation therapy (IMRT; 60–70 Gy/30–35 fractions to the primary tumor, 54–56 Gy/30–35 fractions to elective lymph nodes), with or without concurrent chemotherapy, as previously described [6]. Concurrent chemotherapy was given as cisplatin 100 mg/m^2^ intravenously infused once every 3 weeks or cisplatin 40 mg/m^2^ given once weekly. Chemotherapy was initiated on the first day of RT. Relevant clinicopathologic data were abstracted from the electronic medical record and kept securely in a Research Electronic Data Capture (REDCap) database [7,8]. Further information regarding our standard institutional management of OM has been described previously [9,10].

### 2.3. Symptom Assessment

Weekly patient evaluations were performed for all patients while undergoing RT through physical exams and patient-reported responses to the Oral Mucositis Weekly Questionnaire-Head and Neck Cancer (OMWQ-HN) survey [11]. On the first day and last day of RT patient-reported quality of life (QOL) was assessed with the European Organization for Research and Treatment of Cancer Quality of Life questionnaire C30 and HNC-specific module (EORTC-QLQ-C30 and H&N35) [12,13]. The EORTC-QLQ-C30 is a 30-item questionnaire composed of multi-item scales and single items that reflect the multidimensionality of the quality-of-life construct [13]. It incorporates five functional scales (physical, role, cognitive, emotional, and social), three symptom scales (fatigue, pain, and nausea and vomiting), a global health and quality of life scale, as well as single items assessing additional symptoms commonly reported by cancer patients (dyspnea, appetite loss, sleep disturbance, constipation, and diarrhea). Sleep disturbance was assessed using one question: “During the past week, have you had trouble sleeping?” with the responses “not at all”, “a little”, “quite a bit”, and “very much”. The EORTC H&N35 includes symptoms of oral pain (4 items), swallowing problems (4 items), sense problems (2 items), speech problems (3 items), trouble with social eating (4 items), trouble with social contact (5 items), and less sexual interest and enjoyment (2 items). Also included are Likert scale single items measuring teeth problems, mouth opening problems, dry mouth, sticky saliva, coughing, and feeling ill. Each symptom scale and Likert scale single item was converted into a score ranging from 0 to 100, according to the EORTC guidelines. A higher score indicates a higher level of symptoms or problems, and a score difference of 10 or greater is considered clinically significant [14]. Figures based on the survey data were generated using Microsoft^®^ Excel^®^ for Microsoft 365 MSO (Version 2205 Build 16.0.15225.20362).

### 2.4. Sleep Questionnaires

Two sleep questionnaires were given on the first day of RT. The Insomnia Severity Index (ISI) is a valid and reliable 7-item questionnaire that is commonly used in clinic settings and research to rate current insomnia severity. ISI scores range from absence of insomnia (0–7), to subthreshold (8–14), moderate (15–21), and severe insomnia (22–28) [15]. The ISI has also been validated in cancer patients [16].

The Sleep Disorders Symptom Checklist-25 (SDS-CL-25) is a 25-item questionnaire designed for screening multiple sleep disorders including insomnia and obstructive sleep apnea (OSA) [17]. Each question is scored as 0 points for a response of “never”, 1 point for “once a month”, 2 points for “1–3 times a week”, 3 points for “3–5 times a week”, and 4 points for “>5 times a week”. A disorder may be deemed appropriate for follow-up and considered a positive screen if one or more endorsements with a category have response option 3 or 4 checked. The severity of each sleep disorder can be obtained by summing the individual item scores for each category and dividing by the number of items to yield an average score per disorder (maximum range 0–4). Cumulative sleep morbidity was calculated as the sum of all of the endorsements across the 25 items (maximum range 0–100).

### 2.5. Statistical Analysis

Descriptive statistics were calculated for patient characteristics, treatment information, and sleep assessments. ISI score was examined as both a continuous measure and categorically. The SDS-CL-25 was examined categorically for OSA screening and continuously for OSA severity and cumulative morbidity scores. Associations between baseline subthreshold or greater insomnia and OSA screen status with selected QOL measures on the first and last day of RT were assessed using the Mann–Whitney U test. Differences between patient cohorts based on insomnia and OSA status were assessed with Mann–Whitney U test for continuous variables and Fisher’s exact test for categorical variables.

To evaluate the impact of baseline insomnia and OSA on OM, the associations between ISI and OSA groupings and longitudinally measured OM outcomes were investigated using linear mixed models (LMMs) with random time effects. The LMMs were fit by restricted maximum likelihood (REML). The statistical significance of each grouping and the time-fixed effects were determined by t-tests using Satterthwaite’s method.

Logistic univariate and multivariate analyses were performed to identify sleep survey factors associated with severe OM development (“quite a lot” or “extreme” reported for the OMWQ-HN mouth and throat soreness item). Patients were grouped in the severe OM category based on their highest reported OM score during RT. Univariate logistic regression was employed to assess for associations between clinicopathologic variables and severe OM. Variables with *p*-values < 0.25 on univariate analysis were then incorporated into the logistic multivariate regression model. Adjusted odds ratios (aOR) and 95% confidence intervals (CI) were calculated for severe OM and each of the outcomes of interest.

To examine the performance of a single sleep item asking about trouble sleeping from the EORTC-QLQ-C30 in terms of categorizing the severity of sleep problems, we calculated percent agreement, Cohen’s kappa in comparison with ISI categories, and Fisher’s exact test. Two-sided *p* < 0.05 was considered statistically significant. All analyses were performed using R (version 4.2.1, R Project for Statistical Computing, Vienna, Austria). Data analysis was performed from May 2023 to June 2023.

## 3. Results

A total of 87 patients were included in this study. The median age was 64.6 (IQR, 57.7–70.4) and 70 patients were male (80%). Most patients had tumors of the oropharynx (51%) and received concurrent chemotherapy with radiation (83%). The majority of patients were former smokers (54%) with an ECOG performance status of 0–1 (83%). Table 1 contains complete patient demographic and treatment information.

### 3.1. Insomnia and Obstructive Sleep Apnea

Patient sleep outcomes are shown in Table 2. The median ISI score was 6.0 (IQR, 2.0–11.5), with 53 patients (61%) categorized as having no insomnia, 20 patients (23%) with subthreshold insomnia, 11 patients (13%) with moderate insomnia, and 3 patients (3%) with severe insomnia. There were 47 patients (54%) who screened positive for OSA with a median OSA severity score of 0.8 (IQR, 0.3–1.6). Only eight patients had a documented medical history of OSA, of whom seven were included among those who screened positive for OSA. Eight patients reported daily continuous positive airway pressure (CPAP) device use on the SDS-CL-25 survey, with three of these patients overlapping with those who had a documented OSA diagnosis. The median cumulative sleep morbidity score was 15.0 (IQR, 10.0–29.5).

### 3.2. Quality of Life

Patients who screened positive for OSA had statistically significantly worse xerostomia (*p* < 0.001), fatigue (*p* < 0.001), pain (*p* = 0.01), and insomnia (*p* = 0.003) at the start of RT (Table 3) compared to patients that screened negative for OSA. Upon completion of RT, those screening positive for OSA had worse physical function (*p* = 0.01), sticky saliva (*p* = 0.02), fatigue (*p* = 0.007), insomnia (*p* = 0.009), and pain (*p* = 0.005) compared to those screening negative for OSA. There were no differences in patient demographics or treatment between those who screened positive and those who screened negative for OSA (Supplementary Table S1). Among the eight patients reporting CPAP use, mean xerostomia scores were lower at the start and end of radiation compared to those screening positive for OSA without CPAP use (scores of 25 and 46, respectively).

### 3.3. Oral Mucositis

**Figure 1:**
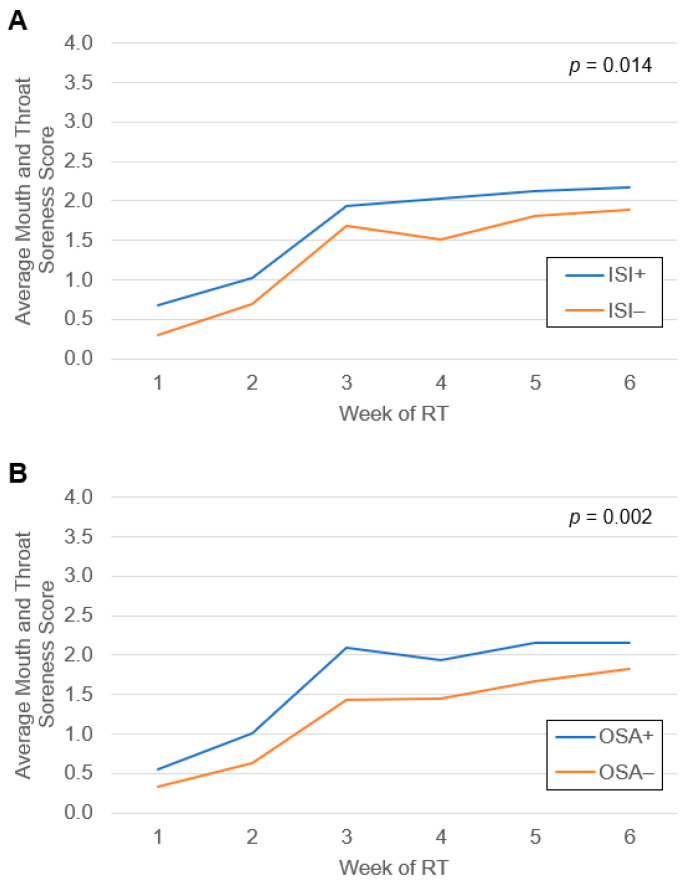
Average mouth and throat soreness scores from patient-reported responses to the Oral Mucositis Weekly Questionnaire-Head and Neck Cancer survey during radiation therapy for head and neck cancer grouped by (**A**) baseline subthreshold or greater insomnia score on the Insomnia Severity Index (ISI) and (**B**) baseline obstructive sleep apnea (OSA) screen status. Differences between groups were assessed by linear mixed models.

A total of 51 patients (59%) developed severe OM during treatment. Average weekly mouth and throat soreness scores during RT grouped by subthreshold or greater insomnia score on the ISI and by OSA screen status are shown in Figure 1. Upon linear mixed model evaluation, both subthreshold or greater insomnia (*p* = 0.01) and positive OSA screen (*p* = 0.002) were associated with higher mouth and throat soreness scores compared to those without insomnia or negative OSA screen, respectively. Univariate Cox regression identified several factors that met the threshold for inclusion in the multivariate model, which comprised sex, primary tumor site, clinical stage, HPV status, and smoking status (Supplementary Table S3). Upon logistic MVA (Table 4), the development of severe OM was significantly associated with ISI subthreshold or greater insomnia (aOR 3.36 [1.07–11.55], *p* = 0.04) and trended towards significance for OSA severity score (aOR 1.87 [1.05–3.74], *p* = 0.05) and cumulative sleep morbidity score (aOR 1.04 [1.00–1.09], *p* = 0.05). Positive screen for OSA and total ISI score were not associated with the development of severe OM.

### 3.4. Agreement of Sleep Assessment between EORTC-QLQ-C30 and ISI

Trouble sleeping at baseline assessed via the EORTC questionnaire was associated with trouble sleeping at baseline assessed via ISI questionnaires (*p* < 0.001; Table 5). Patients who reported quite a bit or very much trouble sleeping were more likely to report more severe insomnia. The percent agreement between categories of the two sleep assessments was 49% and the Cohen’s kappa was 0.47, indicating moderate agreement.

## 4. Discussion

This investigation of sleep disturbances and symptom burden in patients with HNC undergoing RT or CRT yielded several novel findings. First, both baseline subthreshold or greater insomnia scores on the Insomnia Severity Index (ISI) and baseline obstructive sleep apnea (OSA) screen status are common and significantly impacted the average patient-reported mouth and throat soreness scores during all weeks of radiation therapy. Second, both baseline insomnia and positive OSA screen predicted significantly worse QOL across multiple domains at the beginning and end of treatment. Third, baseline ISI was significantly associated development of severe oral mucositis. Fourth, the EORTC trouble sleeping question was significantly correlated with ISI. These findings underscore the importance of sleep evaluation for this patient population to target sleep improvement as a method of mitigating RT symptom burden.

Baseline insomnia was identified in 39% of patients using the ISI survey, which is similar to what has been previously reported for patients with HNC. In a systematic review of sleep disturbances in HNC patients, Santoso et al. have shown the prevalence of insomnia to be 29% before treatment and 45% during treatment [18]. This study also noted baseline hypersomnolence and sleep-related breathing disturbances were prevalent in this population, with rates of 16% and 66%, respectively [18]. Notably, our study found no difference in EORTC insomnia score from baseline to end of RT for the entire cohort (35.2 and 33.7, respectively). Another study has demonstrated 44% of HNC patients have poor sleep quality before treatment, with female sex identified as an associated factor which was similarly found in our study [19].

OSA screen was positive in 54% of patients. Consistent with our findings, a systematic review of OSA in HNC patients has shown the baseline incidence of OSA in this population to be 60% [20]. The incidence of poor sleep quality in the general population is 27–36%, suggesting that HNC patients may be at higher risk for insomnia [21,22]. These findings highlight the high prevalence of sleep disturbances in HNC patients. Given this prevalence of and dramatic association of baseline sleep disturbances with OM and QOL outcomes, clinical trials of HNC patients treated with RT should consider documenting sleep measures.

Remarkably, ISI subthreshold or greater insomnia patients had worse QOL across all domains at the start of RT. This finding persisted for most patients upon treatment completion. While the data are conflicting, both female sex [23,24,25] and smoking [26,27,28] have been implicated in worse OM. For patients screening positive for OSA, worse QOL was seen at treatment start and completion for fatigue, pain, and insomnia. This is consistent with a previous study that showed patients with OSA have a reduction in QOL proportional to severity, including the domains of bodily pain, vitality, and physical functioning [29].

Interventions to improve sleep quality in HNC patients may have the potential to delay the onset of OM pain. Sleep deprivation has the potential to elicit hyperalgesic changes that can increase pain sensitivity and vulnerability to pain, causing a vicious cycle whereby worsening pain makes sleep more difficult [30]. Supporting this, a large population-based study has shown that the frequency and severity of insomnia are associated with pain sensitivity in a dose-response manner [31].

Though over half of the patients screened positive for OSA, fewer than 10 percent were actually being treated with continuous positive airway pressure (CPAP). Exploratory analysis of the patients using CPAP found that they had lower xerostomia scores than those screening positive for OSA. Adherence to CPAP therapy in patients with OSA has been linked to improved QOL and patient symptoms including dry mouth upon awakening, excessive fatigue, and decreased energy [32,33]. Treatment of OSA via CPAP with humidification may have the additional benefit for patients with HNC undergoing RT of directly benefitting mucositis by improving mucosal hydration [34].

There was significant agreement for trouble sleeping at baseline assessed via the EORTC and ISI questionnaires. Patients who reported quite a bit or very much trouble sleeping were more likely to report clinically significant insomnia with moderate agreement. These findings indicate that the EORTC question is an excellent screening question to determine if the ISI should be administered. Omitting the ISI in patients who answered “Not at all” to the EORTC sleeping item would have failed to detect only 3 out of 34 patients with subclinical or greater insomnia, with these 3 missed patients having subclinical insomnia. To minimize the survey burden in clinics or clinical trials, the EORTC item could be used to screen patients to receive the full ISI. The ISI is well correlated with a similar single item from the Patient Health Questionnaire-9 survey [35].

The strengths of this study include the use of validated sleep questionnaires (ISI and SDS-CL-25), longitudinal collection of QOL and oral pain data, comprehensive adjustment of potential confounding factors, and being the first to examine the relationship between sleep and OM pain in HNC patients undergoing RT. This study is limited by sample size and lack of objective sleep data (through actigraphy or formal sleep monitoring) to analyze alongside the patient-reported data.

## 5. Conclusions

Insomnia and OSA are highly prevalent in patients with HNC undergoing RT. These sleep disturbances are associated with worse QOL and OM during treatment. Documentation and treatment of baseline insomnia and OSA should be considered in clinical trials of OM in HNC.

## Figures and Tables

**Table 1:** Patient demographic and treatment information.

Variable	No. (%)
Median age, y (IQR)	64.6 (57.7–70.4)
Sex	
Male	70 (80)
Female	17 (20)
Race	
White	78 (90)
Black	2 (2)
Native American	2 (2)
Unknown	5 (6)
Ethnicity	
Not Hispanic or Latino	81 (93)
Hispanic or Latino	2 (2)
Unknown	4 (5)
ECOG	
0	48 (55)
1	24 (28)
2	15 (17)
Median BMI (IQR)	28.8 (24.4–32.8)
Primary Site	
Oropharynx	44 (51)
Hypopharynx	3 (3)
Larynx	15 (17)
Oral cavity	12 (14)
Parotid gland	4 (5)
Unknown primary	9 (10)
Stage	
I	26 (30)
II	17 (20)
III	21 (24)
IV	23 (26)
HPV Status	
Positive	46 (53)
Negative	13 (15)
Unknown	28 (32)
Treatment	
RT alone	7 (8)
ICT + CCRT	4 (5)
CCRT	54 (62)
Surgery + RT	8 (9)
Surgery + CCRT	14 (16)
CCRT	
Yes	72 (83)
No	15 (17)
Neck RT	
Unilateral	19 (22)
Bilateral	64 (74)
N/A	4 (5)
Smoking Status	
Never	28 (32)
Former	47 (54)
Current	12 (4)

IQR: interquartile range; ECOG: Eastern Cooperative Oncology Group performance status; BMI: body mass index; HPV: human papillomavirus; RT: radiation therapy; ICT: induction chemotherapy; CCRT: concurrent chemoradiation.

**Table 2:** Selected patient sleep questionnaire results.

Item	No. (%)
ISI	
No Insomnia	53 (61)
Subthreshold Insomnia	20 (23)
Moderate Insomnia	11 (13)
Severe Insomnia	3 (3)
Median ISI Score, IQR	6.0 (2.0–11.5)
OSA Screen	
Positive	47 (54)
Negative	40 (46)
Median OSA Severity Score, IQR	0.8 (0.3–1.6)
Median Cumulative Sleep Morbidity Score, IQR	15.0 (10.0–29.5)

ISI: Insomnia Severity Index; IQR: interquartile range; OSA: Obstructive sleep apnea.

**Table 3:** Associations between screening positive for obstructive sleep apnea (OSA) and insomnia with selected quality of life (QOL) metrics in patients with head and neck cancer at the start and completion of radiation treatment (RT), which were assessed using the Mann–Whitney U test.

	Mean QOL Score			Mean QOL Score		
	OSA Screen Positive (*n* = 47)	OSA Screen Negative (*n* = 40)	*p*-Value	Insomnia Present * (*n* = 34)	Insomnia Not Present (*n* = 53)	*p*-Value
**RT Start**						
Global function	62.2	70.0	0.10	54.7	73.0	<0.001
Physical function	80.7	87.0	0.15	74.1	89.7	<0.001
Fatigue	61.0	80.3	<0.001	51.0	82.0	<0.001
Pain	39.7	23.3	0.01	49.5	21.1	<0.001
Insomnia	45.4	23.3	0.003	63.7	17.0	<0.001
Xerostomia	40.4	8.3	<0.001	41.2	15.7	<0.001
Sticky saliva	29.1	16.7	0.25	41.2	11.9	<0.001
**RT Completion**						
Global function	52.8	59.2	0.17	53.4	57.2	0.40
Physical function	69.2	82.5	0.01	68.0	80.0	0.005
Fatigue	45.2	59.4	0.007	42.8	57.4	0.01
Pain	48.2	32.9	0.005	48.5	36.5	0.06
Insomnia	43.3	22.5	0.009	51.0	22.6	<0.001
Xerostomia	60.3	49.2	0.15	57.8	53.5	0.58
Sticky saliva	75.9	60.8	0.02	81.4	61.0	0.002

* Patients who scored as having subthreshold or greater insomnia on the ISI had worse QOL across all selected domains at the start of RT (*p* < 0.001; Table 3). Upon completion of RT, patients with ISI subthreshold or greater insomnia continued to have worse physical function (*p* = 0.005), fatigue (*p* = 0.01), insomnia (*p* < 0.001), and sticky saliva (*p* = 0.002) compared to patients without insomnia. Patients with subthreshold or greater insomnia were more likely to be female (*p* = 0.005) and a former or current smoker compared to patients without insomnia (*p* = 0.04; Supplementary Table S2).

**Table 4:** Logistic multivariable analysis for clinical characteristics associated with the development of severe oral mucositis.

Characteristic	aOR (95% CI) *	*p*-Value
Sex		
Male	1 [Reference]	
Female	2.12 (0.47–12.27)	0.35
Site		
Oropharynx	1 [Reference]	
Hypopharynx	0.35 (0.01–10.71)	0.56
Larynx	1.22 (0.13–11.95)	0.86
Oral cavity	1.82 (0.18–19.79)	0.61
Parotid gland	0.54 (0.02–13.80)	0.71
Unknown primary	2.52 (0.43–17.69)	0.32
Stage		
I	1 [Reference]	
II	7.66 (1.66–44.17)	0.01
III	3.39 (0.78–16.17)	0.11
IV	3.02 (0.56–17.50)	0.20
HPV Status		
Negative	1 [Reference]	
Positive	4.86 (0.44–61.16)	0.20
Unknown	3.80 (0.73–21.59)	0.12
Smoking		
Never	1 [Reference]	
Former	1.25 (0.33–4.96)	0.75
Current	2.60 (0.40–19.11)	0.32
ISI Category		
None	1 [Reference]	
Subthreshold or greater	3.36 (1.07–11.55)	0.04
ISI Score	1.05 (0.97–1.16)	0.25
OSA Screen		
Negative	1 [Reference]	
Positive	2.34 (0.81–7.22)	0.12
OSA Severity Score	1.87 (1.05–3.74)	0.05
Cumulative Sleep Morbidity Score	1.04 (1.00–1.09)	0.05

aOR: adjusted odds ratio; CI: confidence interval; HPV: human papillomavirus; ISI: Insomnia Severity Index; OSA: obstructive sleep apnea. * The multivariate model was adjusted for sex, disease site, smoking status, clinical stage, and HPV status.

**Table 5:** Agreement between baseline ISI clinical insomnia categories and the trouble sleeping item from the EORTC QLQ-C30.

	EORTC Response			
ISI Category	Not at All (*n* = 33)	A Little (*n* = 27)	Quite a Bit (*n* = 16)	Very Much (*n* = 11)
None (*n* = 53)	30	20	2	1
Subclinical (*n* = 20)	3	7	9	1
Moderate (*n* = 11)	0	0	4	7
Severe (*n* = 3)	0	0	1	2

Percent agreement: (30 + 7 + 4 + 2)/(87) = 49%; kappa = 0.47; Fisher’s exact test *p*-value is <0.001. ISI: Insomnia Severity Index; EORTC QLQ-C30: European Organization for the Research and Treatment of Cancer Quality of Life Questionnaire.

## Data Availability

Research data are stored in an institutional repository and will be shared upon request to the corresponding author.

## Supplementary Materials

The following supporting information can be downloaded at: https://www.mdpi.com/article/10.3390/cancers16071335/s1, Table S1: Patient characteristics of patients screening positive and negative for OSA, Table S2: Patient characteristics of those scored as having subthreshold or greater insomnia on the Insomnia Severity Index at the start of RT, Table S3: Univariate Cox regression of clinicopathologic factors associated with the development of severe oral mucositis.
