# Supplementary material for: Association of Insomnia and Obstructive Sleep Apnea with Worse Oral Mucositis and Quality of Life in Head and Neck Cancer Patients Undergoing Radiation Therapy
Source: Cancers (Basel). 2024 Mar 29;16(7):1335. doi: 10.3390/cancers16071335 (PMC11011024; doi:10.3390/cancers16071335)
Supplement: Supplementary file 1 [file cancers-16-01335-s001.zip › cancers-2916194-supplementary.pdf]

Supplementary Table S1: Patient characteristics of patients screening positive and negative for OSA. Comparisons were made using Fisher's exact test.

	**OSA Screen Positive (n=47)**	**OSA Screen Negative (n=40)**	
	**No. (%)**	**No. (%)**	**P-value**
Age, median (IQR), y	62.0 (57.1-69.3)	65.6 (59.4-72.6)	0.22
Sex			
Male	35 (74)	35 (88)	0.18
Female	12 (26)	5 (13)	
Race			
White	45 (96)	33 (83)	0.13
Black	0 (0)	2 (5)	
Native American	1 (2)	1 (3)	
Unknown	1 (2)	4 (10)	
Ethnicity			
Not Hispanic or Latino	44 (94)	37 (93)	0.29
Hispanic or Latino	2 (4)	0 (0)	
Unknown	1 (2)	3 (8)	
ECOG			
0	26 (55)	22 (55)	1.00
1	13 (28)	11 (28)	
2	8 (17)	7 (18)	
BMI, median (IQR)	30.7 (25.2-33.2)	27.9 (24.1-31.6)	0.37
Primary Site			
Oropharynx	25 (53)	19 (48)	0.73
Hypopharynx	2 (4)	1 (3)	
Larynx	7 (15)	8 (20)	
Oral cavity	7 (15)	5 (13)	
Parotid gland	3 (6)	1 (3)	
Unknown primary	3 (6)	6 (15)	
Stage			
I	15 (32)	11 (28)	0.58
II	11 (23)	6 (15)	
III	11 (23)	10 (25)	
IV	10 (21)	13 (33)	
HPV Status			
Positive	26 (55)	20 (50)	0.91
Negative	7 (15)	6 (15)	

Unknown	14 (30)	14 (35)	
Treatment			
RT alone	3 (6)	4 (10)	0.52
ICT + CCRT	1 (2)	3 (8)	
CCRT	31 (66)	23 (58)	
Surgery + RT	3 (6)	5 (13)	
Surgery + CCRT	9 (19)	5 (13)	
CCRT			
Yes	41 (87)	31 (78)	0.27
No	6 (13)	9 (23)	
Neck RT			
Unilateral	11 (23)	8 (20)	0.10
Bilateral	36 (77)	24 (60)	
N/A	0 (0)	4 (10)	
Smoking Status			
Never	13 (28)	15 (38)	0.24
Former	25 (53)	22 (55)	
Current	9 (19)	3 (8)	

IQR: interquartile range; ECOG: Eastern Cooperative Oncology Group performance status; BMI: body mass index; HPV: human papilloma virus; RT: radiation therapy; ICT: induction chemotherapy; CCRT: concurrent chemoradiation.

Supplementary Table S2: Patient characteristics of those scored as having subthreshold or greater insomnia on the Insomnia Severity Index at the start of RT. Comparisons were made using Fisher's exact test.

	**Insomnia Present[*] (n=34)**	**Insomnia Not Present (n=53)**	
	**No. (%)**	**No. (%)**	**P-value**
Age, median (IQR), y	61.4 (56.9-68.2)	66.3 (58.3-72.5)	0.11
Sex			
Male	22 (65)	48 (91)	0.005
Female	12 (35)	5 (9)	
Race			
White	30 (88)	48 (91)	0.54
Black	0 (0)	2 (4)	
Native American	1 (3)	1 (2)	
Unknown	3 (9)	2 (4)	
Ethnicity			
Not Hispanic or Latino	31 (91)	50 (94)	0.83
Hispanic or Latino	1 (3)	1 (2)	
Unknown	2 (6)	2 (4)	
ECOG			
0	15 (44)	33 (62)	0.13
1	10 (29)	14 (26)	
2	9 (26)	6 (11)	
BMI, median (IQR)	28.0 (22.3-33.0)	29.8 (25.1-32.10)	0.50
Primary Site			
Oropharynx	12 (35)	32 (6)	0.20
Hypopharynx	2 (6)	1 (2)	
Larynx	8 (24)	7 (13)	
Oral cavity	6 (18)	6 (11)	
Parotid gland	1 (3)	3 (6)	
Unknown primary	5 (15)	4 (8)	
Stage			
I	9 (26)	17 (32)	0.26
II	6 (18)	11 (21)	
III	6 (18)	15 (28)	
IV	13 (38)	10 (19)	
HPV Status			
Positive	6 (18)	7 (13)	0.23

Negative	14 (41)	32 (60)	
Unknown	14 (41)	14 (26)	
Treatment			
RT alone	2 (6)	5 (9)	0.33
ICT + CCRT	1 (3)	3 (6)	
CCRT	19 (56)	35 (66)	
Surgery + RT	9 (26)	5 (9)	
Surgery + CCRT	3 (9)	5 (9)	
CCRT			
Yes	5 (15)	10 (19)	0.77
No	29 (85)	43 (81)	
Neck RT			
Unilateral	8 (24)	11 (21)	0.78
Bilateral	24 (71)	40 (75)	
N/A	2 (6)	2 (4)	
Smoking Status			
Never	6 (18)	22 (42)	0.04
Former	21 (62)	26 (49)	
Current	7 (21)	5 (9)	

IQR: interquartile range; ECOG: Eastern Cooperative Oncology Group performance status; BMI: body mass index; HPV: human papilloma virus; RT: radiation therapy; ICT: induction chemotherapy; CCRT: concurrent chemoradiation.

*Patients scored as having subthreshold or greater insomnia on the Insomnia Severity Index at the start of RT.

Supplementary Table S3: Univariate Cox regression of clinicopathologic factors associated with the development of severe oral mucositis.

**Variable**	**p-value**
Age	0.94
Sex	
Male	Reference
Female	0.10
Race	
White	Reference
Black	0.99
Native American	0.99
Unknown	0.94
Ethnicity	
Not Hispanic or Latino	Reference
Hispanic or Latino	0.82
Unknown	0.51
ECOG	
0	Reference
1	0.61
2	0.80
BMI	0.31
Primary Site	
Oropharynx	Reference
Hypopharynx	0.21
Larynx	0.49
Oral cavity	0.86
Parotid gland	0.51
Unknown primary	0.71
Stage	
I	Reference
II	0.03
III	0.31
IV	0.11
HPV Status	
Positive	Reference
Negative	0.51
Unknown	0.19
Treatment	

RT alone	Reference
ICT + CCRT	0.99
CCRT	0.94
Surgery + RT	0.75
Surgery + CCRT	0.78
CCRT	
Yes	Reference
No	0.65
Neck RT	
Unilateral	Reference
Bilateral	0.91
N/A	0.77
Smoking Status	
Never	Reference
Former	0.74
Current	0.21
ISI Category	
None	Reference
Subthreshold or greater	0.008
ISI Score	0.02
OSA Screen	
Negative	Reference
Positive	0.14
OSA Severity Score	0.04
Cumulative Sleep Morbidity Score	0.003

ECOG: Eastern Cooperative Oncology Group performance status; BMI: body mass index; HPV: human papilloma virus; RT: radiation therapy; ICT: induction chemotherapy; CCRT: concurrent chemoradiation; ISI: Insomnia Severity Index; OSA: obstructive sleep apnea.
